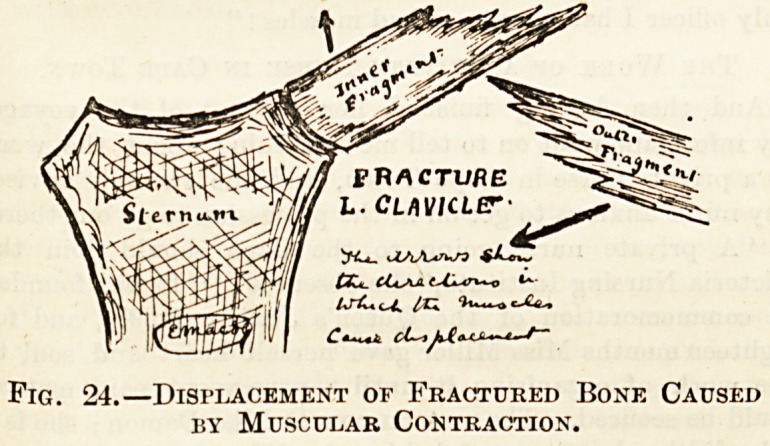# "The Hospital" Nursing Mirror

**Published:** 1900-09-01

**Authors:** 


### "The Hospital" Nursing Mirror.

Being the Nursing Section of "The Hospital."

[Contributions for this Section of "The Hospital" should be addressed to the Editor, The Hospital, 28 & 29, Southampton Street, Strand, London, W.C., and should have the word "Nursing" plainly written in left-hand top corner of the envelope.]

### Notes on News from the Nursing World.

### CONVALESCENT OFFICERS' QUARTERS AT CAPE TOWN.

A member of the Army Nursing Service Reserve, who was sent by the authorities to nurse a case of typhoid at Claremont Sanatorium, writes to us as follows:—

"This sanatorium is managed by Americans, who for some occult reason call the place a 'sanitarium.' The building is a large one, situated on the Glate, a stretch of ground of many miles area at the foot of Table Mountain, covered with grey salt sand, in which pines and heather grow well. We are less directly under the mountain than Cape Town, and the air is consequently better and fresher. One wing containing over fifty beds, mostly in separate rooms, has been taken over by the Government as a convalescent home for officers, who seem to greatly appreciate the comforts provided for them, and better still, to benefit by them. Convalescents only are taken, the typhoid case I nursed being accidental. The patient I had was sent here convalescing from malaria developed typhoid, when it was of course impossible to move him elsewhere. On my arrival I was met by Sister Gray, who is senior in the Army Nursing Service, and wears the medals of five campaigns. There is one civil sister here on day duty; I took the night. Our rooms are all together, with a very nice little sitting-room with sofa, writing table, &c., not far distant. We mess at the staff table, and there, as a rule, the R.A.M.C. men congregate. Things are worked on a very liberal scale, and our menus compare, so all who have tried both say, favourably with those of the best hotels in Cape Town. Certainly, we have an excellent chef, and men who come here looking perfect wrecks pick up wonderfully, and sometimes in a week's time are able to go back to the front again, or to start on the voyage home with a good heart."

### ARMY SISTERS NEEDED AT JOHANNESBURG.

In a letter written to Sir James Blyth, Mr. Alfred Fripp signifies that the 36 nurses who were recently sent out to the Imperial Yeomanry Hospital at Deelfontein are badly needed at Johannesburg. Mr. Fripp says, "We have now to turn the patients out of one of our huts so as to find sleeping accommodation for the 36 sisters and eight wardmaids, while the 80 orderlies are housed in the tents usually used by convalescent patients, and room is made for the nine doctors in some of the other tents. As it is such a long time since we have taken in any large batch of acute cases, the wards throughout the hospitals are what is known technically as light. A couple of months ago the work was so heavy that we should have been glad to have had these 36 new sisters as reinforcements, but really now there is not enough work to occupy our own 48, and it is very much to be hoped that the new arrivals will, without much waste of time, be got on to Johannesburg."

### MONTHLY NURSES AT CAPE TOWN.

One of the sisters on board the s.s. "Gascon" is in England for the first time, being a British Colonial from Cape Town. She is making the most of the time before returning, and has quite a genius for finding her way about London. She has been to the top of the Monument—which is more than many Londoners can say—and has visited the Royal Exchange, the Bank, and Billingsgate Fish Market. She speaks of the kindness of people she meets in giving her directions, and says that they are always ready for a chat when they know they are speaking to a Colonial. She is a monthly nurse in Cape Town, and has been at work there for six years. The certificate she holds is from the Free Dispensary, and she states that her difficulty is not to get work but to avoid being overworked. She had an uneventful voyage on the "Gascon," under Nursing Sister Ward, sister-superintendent.

### THE UP-COUNTRY NURSING ASSOCIATION.

The annual report of the Up-country Nursing Association has been sent to us by the honorary secretary at Naini Tal. It is from many points of view satisfactory, but the committee express anxiety to increase their staff, and mention that, owing to the want of nurses, "nearly as many cases were refused as were attended." The work done, however, has been excellent, and it is freely admitted that the call for subscriptions and donations was very generously responded to. The names of six nurses are given, and it speaks well for the manner in which the association is managed that two of these only left on the expiry of the five years for which they joined. The number of cases attended by the staff in 1899 was 44, and the number of days they put in an attendance was upwards of a thousand. We learn from the report that "the excellent work of the nurses is appreciated by both medical men and patients."

### LADY GORMANSTON AND NURSING AT HOBART.

Prior to the departure of Lady Gormanston from the antipodes, the wife of the late governor of Tasmania presided at the annual meeting of the Hobart District Nursing Association, and in the course of the proceedings a letter of farewell was read to her from the association, in which graceful allusion was made to the sympathy and interest she has shown in nursing work during her residence in the colony. Lady Gormanston, in acknowledging the compliment, confessed that she should have liked to have left the association in a more flourishing condition than it is, and it appears from the statement of Miss A. L. Chapman that, but for the £27 received from the theatricals got up for its benefit by the governor's wife, the members of the association would have had to consider whether they could afford to carry on during the present year. Miss Chapman naturally spoke with some bitterness on this point. "Every year," she said "hundreds—thousands—were sent from Tasmania to help various missions, and bishops and clergy preached the mission cause, while men, women, and children gave money, time, and thought to swell the mission funds. Here in Hobart, men, women, and children, sick, poor, and helpless, were lingering on in dreary pain, and under one hundred pounds a year was all that could be scraped together to help them. Surely Mrs. Jellyby still flourished on this side of the line." The contrast is discouraging, and though Canon Shoobridge, one of the supporters of the nursing association, rightly deprecated the idea of home work and foreign work being placed in opposition, it may fitly be contended in respect to the former that it ought not, in any circumstances, to be left undone.

### "FRICTION" AT THE GUILDFORD WORKHOUSE.

None too soon it has been decided that the House Committee of the Guildford Board of Guardians shall make full inquiry into the relations existing between certain officials. The resignation of a nurse because she was refused by the master leave of absence in a case of necessity might be passed over, especially if, as it is stated, the request was the third made in three weeks after she commenced her duties. But a condition of affairs which, as Mr. Bullen, a member of the Board, says, has resulted in the master and the superintendent nurse holding no verbal communication with each other, must require to be altered. At present, according to Mr. Bullen, any communication between these two officials is done "by notes on pieces of paper." It is clearly the duty of the Guildford Guardians to lose no time in placing things on a better footing than this.

### MONKWEARMOUTH AND SOUTHWICK HOSPITAL.

In the twenty-seventh annual report of the Monkwearmouth and Southwick Hospital the Executive Committee attribute the reduction in the housekeeping accounts to the careful supervision of the contracts by the Ladies' Visiting Committee, assisted by the excellent management of the matron. It is added that "the Ladies' Visiting Committee are of considerable service to the institution," and "that much of the credit for the general condition of the hospital is due to the interest taken in it by these ladies." The matron, Miss Deakin, and the nursing staff are also complimented in the report for their devoted assistance to the medical staff, and it is observed that "not a little of the credit for the successful results is owing to skilful and careful nursing."

### THE GIFT TO THE PORTADOWN NURSING SOCIETY.

It will be remembered that Colonel Saunderson, M.P., was so convinced of the necessity of providing an ambulance for the Portadown District Nursing Society that he offered to present them with one. The welcome gift has now been received, and the society are in possession of an ambulance litter of the "Viaduct" pattern, which bears on the front a brass plate with the following inscription: "Presented by the Right Hon. Colonel and Mrs. Saunderson to the Portadown District Nursing Society, August, 1900." We hope that the example set by the gallant member will be followed in other cases. All nursing societies should have an ambulance of their own, but unfortunately many cannot afford to purchase one. The want can therefore often only be met by private generosity.

### CHESTERFIELD VICTORIA HOME FOR NURSES.

At the annual meeting of the subscribers to the Chesterfield Victoria Home for Nurses it was stated that during the past year applications for nurses had considerably increased in number, and that cases had seldom been refused, save in the early part of the year, when, owing to the influenza epidemic, as many as four a day were often turned away. A feature of the report was a statement from the medical officer of the North Derbyshire Hospital expressing the indebtedness of his committee to the matron and nurses of the Home for their assistance in the temporary hospital at the time of a typhoid outbreak in Shirebrook. In addition to the attendances for which fees were paid, no less than 60 cases, involving 834 visits, were nursed gratuitously, a fact which is highly creditable to the institution.

### THE DANGERS OF CELLULOID COLLARS.

Amongst the necessities for a nurse proceeding to South Africa celluloid collars and cuffs have always been reckoned one of the most important, owing to the difficulty of obtaining good laundresses. For the sake of those nurses on foreign service, as well as many who may adopt celluloid in preference to linen elsewhere for other reasons, the experience of a motorman on one of the American electric lines is worth repeating. His motor burnt out, and when he attempted to fix it he got the controller charged with electricity. The end happened to touch his celluloid collar, and in a moment there was a quick blaze all round the man's throat. He was seriously burnt, and removed to the nearest hospital in a precarious condition. The highly inflammable character of celluloid is probably not generally known, and it is wise to remove cuffs or collars of this material if there is even the remotest chance of their coming in contact with the flames of a fire, gas, or electric light.

### A SYRIAN WARDMAN.

"Our native wardman, or orderly," writes a correspondent, "is a great character. Though by reason of his Eastern manners and light of looking at things Selim is often very exasperating, I believe he has our real interests at heart. In his peculiar way he is much attached to the nurses. It gives him much pleasure and satisfaction to escort either of us into the town for shopping, or for a short journey to pay a call. A little while ago it was necessary for me to visit the native doctor's wife, who lives about a mile from the hospital. Selim accompanied me to the house, and much astonished me by coming into the drawing-room, where he sat down beside me upon a divan. When the lady appeared, Selim, in his most patronising tones, introduced me. Instead of giving their visitors tea in the afternoon, women of the Syrian upper classes have coffee, wine, and sweetmeats brought in. When the tray was handed round I was dismayed to see Selim coolly help himself! The whole incident was most amusing, especially as I did not need his services as interpreter, because the doctor's wife spoke to me in French."

### SHORT ITEMS.

The annual report of the Levenshulme District Nursing Association shows a small balance on the wrong side, and an appeal has been made to the subscribers to double their contributions. Another nurse is badly wanted.—Miss Mary Pavyer, the new matron at the Scarborough Infectious Diseases Hospital, writes to say that she was trained at the Westminster Hospital, and has been charge nurse and deputy matron of the City Hospital East, Liverpool, for four and a-half years.

### Lectures on Nursing for Probationers.

By E. MacDowel Cosgrave, M.D., &c., Lecturer to the Dublin Metropolitan Technical School for Nurses.

### XIV.—MOVEMENT.

There are two classes of muscles in the body. One class is the red muscles, which compose the flesh; these are called voluntary muscles, because they obey the will—these are sometimes called skeletal, because they are attached to bones. The other class is the pink, involuntary muscles, such as are found in the walls of the digestive canal; these are independent of the will, and so act during sleep as well as when waking. The heart is composed of an intermediate kind of muscle; it is red and strong, like the skeletal muscles, but acts without direction of the will.

The voluntary muscles are attached to the periosteum covering the bone, either directly by their fibres or by tendons from which their fibres spring. Tendons are glistening white in appearance, and immensely strong, much stronger than the bodies of muscles, so they take up less space and are generally used in the neighbourhood of joints which would be thick and clumsy if the fleshy muscles passed over them. Thus, at the wrist the tendons take up little room, and allow free play to the joints, yet they act on the fingers with the full power of the bulky muscles in the upper part of the fore-arm.

The terms "origin" and "insertion" are applied to the places where the ends of the muscle are attached; the origin is the most fixed end, the insertion is the most movable end, thus the muscles whose tendons pass over the wrist have

Fig. 24.—Displacement of Fractured Bone Caused by Muscular Contraction.

their origin in the fore-arm, their insertion in the fingers. The term extensors and flexors are used to divide the muscles of the limbs into groups; those that straighten out joints are called extensors, those that bend joints are called flexors.

It is only possible here to mention the chief groups of muscles, naming only the most important; many muscles which help in the chief movements are omitted, as are many of the less prominent movements.

The pectoral muscles on the front of the chest move the arm forward, the muscles of the side of the neck raise the shoulders, and the deltoid which covers the point of the shoulder lifts the arm out from the side. The muscles of the back move the scapula and arm.

In the arm, the biceps in front raises the fore-arm flexing the elbow, the triceps at the back extends it. In the fore-arm the bodies of the muscles are chiefly at the upper part, their tendons running to the fingers. The muscles at the front are flexors, those at the back are extensors. The ball of the thumb contains muscles which give the thumb so many more movements than the fingers.

The thigh is flexed by the psoas which lies at the back of the abdominal cavity; it is extended by the glutens which forms part of the mass of the buttock. The four muscles of the front of the thigh have a common tendon which is attached to the patella, which again is attached to the front of the tibia by a continuation of the ligament. These four muscles extend the knee-joint, the muscles at the back of the thigh flex it. The muscles inside the thigh draw the knee in, crossing one leg over the other.

The two large muscles whose bodies form the calf of the leg are attached to the heel by the tendo Achilles; the contraction of these muscles raises the heel from the ground in walking. The muscles in front between the tibia and fibula draw up the front of the foot and toes. The foot has strong muscles, and its arch is strengthened by ligaments.

The head is moved by the muscles of the back and neck. The deeper muscles of the back are immensely strong; they raise the body from the stooping position, support it in the erect position, and enable heavy weights to be carried in front.

The abdominal organs are supported at the sides by three layers of muscle; in the front the right and left rectus muscles run from the lower end of the sternum to the pubes.

Muscles are naturally in a state of contraction; this keeps the bones of the joints well pressed together, and enables the erect position to be maintained without effort. This contracture causes deformity in dislocation and in fracture. (Fig. 24.)

In paralysis the affected muscles ceasing to act the other muscles pull the part over; thus in facial paralysis the face is pulled to the unaffected side.

Unused muscles waste, as is seen in fractured limbs that have been kept some time in splints; so in weakness of muscles we should not aim at artificial support, but at strengthening by exercise and massage.

### Presentations.

Sherburn Hospital, Durham.—A series of very interesting presentations took place at the Sherburn Hospital a few days ago. Miss Rhoda Simpson, who has lately resigned the post of matron and is commencing a home for a limited number of paying guests and invalids requiring a certain amount of skilful attention at Grange-over-Sands, Lancaster, received from the nurses and maids a silver salver with a suitable inscription, wishing her success in her new undertaking. The male patients (past and present) gave her a beautiful oak pedestal writing table with a silver plate attached. The female patients also presented her with a dinner service and afternoon tea set of blue Queen Anne china. After the several presentations Miss Simpson thanked the donors for their beautiful gifts, and assured them that she would never forget the happy days she had spent at Sherburn Hospital. Miss Simpson was also the recipient of a handsome testimonial from the master and governors of the institution, who at a special meeting of the board passed a hearty vote of thanks to the matron for her good and faithful service to the sick during her twelve years of service, and asked her to receive as a mark of their appreciation for "duty nobly done" a cheque for £50.—Nurse Marion McKay, who has held the position of charge nurse for some time past, and who is leaving the hospital to join Miss Simpson as nurse in her new home, was also presented at the same time with a handsome Gladstone travelling bag by the master (the Rev. H. A. Mitton) and a silver watch from the matron (Miss Simpson), also a belt with silver buckle from her colleagues, with all geod wishes for her future happiness and success.

Camberwell Infirmary.—On Saturday last Miss Louisa Garrett-Anderson, M.B.B.S.Lond., was presented with a silver bowl by the medical and nursing staff of the Camberwell Infirmary, as a token of their admiration and regard for her. Dr. Anderson has done an excellent year's work at Camberwell, and her resignation is regretted by everyone connected with the infirmary.

### A Nurse at the Red Cross Depot.

By a Correspondent.

"We were most fortunate," said my informant, formerly a Bart.'s nurse, "in having the staff of the Portland Hospital on board the 'Canada.' Their work in South Africa has been splendid; it will come out well when the reports are published."

"Are you a member of the staff?" I inquired.

### At the Red Cross Depôt.

"No, I was a private nurse in Cape Town before my marriage, and up to the time the 'Canada' left I was helping Lady Furley at the Red Cross depôt in Cape Town. It was very hard work, for we had all the unpacking of huge cases to do, as well as the packing up again before the things were sent off to the various hospitals. It meant beginning at half-past nine every morning and working hard up to half-past five, for Lady Furley at least was not among the idle women we have heard so much of."

"Did the parcels usually reach their destination?"

"There was a terrible amount of robbery; although four policemen were on duty at the docks, the cases often arrived nearly empty, and one had to dip down to the bottom for what was left. Strict watch was kept and inquiries made, but the robbery still went on."

"And were things lost on the railway, too?"

"Yes. At last Sir John Furley had everything sealed, and each station master along the line had to examine the cases or parcels and be personally responsible for them. Still things were lost; perhaps one pair of socks would be abstracted from every packet in a huge bale of socks, which showed that it was systematically done."

"Did many ladies help at the depôt?"

"Yes; and Lady Furley herself worked tremendously hard. They were mostly officers' wives, naturally anxious to help. One day in the week was devoted to 'papers,' *i.e.*, the fresh papers from home were put up in piles for the different hospitals. Then two or three mornings were devoted to the kit bags, different ladies taking it in turns to do this, and usually bringing several ladies to help. The packing up of warm clothes and comforts for sick went on all day independently of the other work, and our helpers were constantly changing, some only staying a few weeks, but all anxious to help whilst out there. The Good Hope Society and Red Cross work were worked separately, although the one shed was used a good deal for both."

### On Board the "Canada."

"And now, will you tell me about the voyage?"

"I have already mentioned the staff of the Portland Hospital, and I, personally, was very glad to find not only Dr. Tooth and Mr. Calverley, both Bart.'s men, on board, but also my first sister at Bart.'s, Sister Davies and Sister Russell, both of the Portland; and Mrs. Blackburn, who is a colonial nurse, and myself, made up the nursing staff."

"Did you arrange the work as on land?"

"Not at all, and to me army nursing was something quite strange and new. There was very little work for us; we went down to the hospital after breakfast, say a quarter-past nine, and gave medicines and saw to the dressings. The orderlies were excellent men, belonging to the St. John Ambulance Association. Our great difficulty was in receiving no charts with the patients, and so having no history to guide us. Consequently, when we found a man with a temperature of 103, who vaguely said he had had fever, but couldn't give any definite account of himself, it was often perplexing, and it would have saved time and some anxiety if we had been given charts when the more serious cases came to us, the patients themselves being rather vague as to what had been done for them."

"Where did they mostly come from?"

"From the base hospital at Wynberg, and also from Maitland, Rosebank, and Woodstock."

### A Few of the Patients.

"Were any of the cases very bad?"

"Some were very serious. Several were lame as an effect of typhoid, and one had a mastoid abscess, which is rare. I went down one morning and found one poor fellow, who had seemed to be brightening up during the voyage, quite unhinged; he repeated several times that 'they were conjuring over there,' meaning a group of men playing cards. I told one of the orderlies to watch him very carefully for fear he might attempt to injure himself. There was no telling what he might do in a state of such depression as he was in."

"Did he recover?"

"I cannot say, as this was near the end of the voyage, and he was drafted to Netley."

"Were there many gunshot wounds also?"

"No. One man was shot through the spine, and was paralysed in consequence. Another, whom I found wearing a curiously-twisted bullet which he had put on a ring, told me it came through his brain. 'You see, Sister,' he said, 'there was nothing to stop it, so it came out!' Many ladies are wearing these ghastly souvenirs of this terrible war on their bracelets."

"How many beds were there?"

"Sixty-three, besides the swinging cots, with which we had nothing to do. There were 1,400 men on board. The only officer I had charge of had measles!"

### The Work of a Private Nurse in Cape Town.

And then, having finished her account of the voyage, my informant went on to tell me something about the work of a private nurse in Cape Town, and she strongly advises any nurse anxious to get on in the profession to go out there.

"A private nurse going to the Cape should join the Victoria Nursing Institute," she observed. "It was founded in commemoration of the Queen's Jubilee (1897), and for eighteen months Miss Miller gave herself heart and soul to the work of organising it until a permanent paid matron could be secured. The matron now is Miss Damon; she is a splendid head. One useful thing she did quite lately was to give lessons in bed making, changing sheets, &c., to the ladies of the town, who were anxious to nurse the wounded, and had passed the St. John Ambulance. Some of the nurses gave up their rooms for these lessons, which were most useful."

### Something About the V.N.I.

"How many members has the Institute?" I inquired.

"About fifty; but only thirty are resident. It is an old Dutch house, very comfortable, and everything is beautifully arranged. The idea was to secure for nurses a cheaper way of living than any other the town afforded, but this is very difficult, as provisions are so dear."

"What is the cost of living at the institute?"

"The nurses pay nine shillings a week for a room, and three shillings a day for food; but they only pay for food as they have it. There are no rules except that if they are not going to be in by ten o'clock they must let the matron know. Then there is an entrance fee of one guinea, for it is a club and registration bureau as well as a home, and one shilling in the pound is charged on earnings, for incidental expenses, such as postage, which is, I think, very reasonable."

"What are the qualifications for membership?"

### A Sine Qua Non.

"A three years' certificate for hospital training; no one is admitted without. There is not much chance of work unless a nurse joins the V.N.I., as the doctors for a large area round Cape Town now simply telegraph to the matron when they want a nurse. They are widening the circle tremendously, and are now able to make use of the Johannesburg nurses. That, by the way, is one of the best managed hospitals in South Africa. The matron treats her nurses like women, and not, as so many do, as if they were children. And if a nurse is not a woman at twenty-three, when is she going to be? The V.N.I. is very keen about district nurses, too."

"What does a district nurse earn?"

"Sixty pounds a year clear, with uniform and washing."

"And a private nurse from the V.N.I.?"

"The average rate is three guineas weekly, and all expenses. This is arranged by the nurse herself, independently of the matron. The nurse takes with her a printed slip with particulars of her requirements, such as that half-a-crown a week is to be paid for washing, if not included in board and lodging; and she fixes the sum according to circumstances."

The Question of Uniform.

"Is there a regulation uniform?"

"No, that also is left to individual discretion, though they are talking of having one for all alike. Whatever it is, it must be light; I took out my Galatea dress, but I soon found that it was impossible to work in such heavy clothes, besides the inconvenience of adding to one's luggage."

Finally, my informant told me about the silver medals given by Sir Gordon Sprigg to those nurses who have belonged to the V.N.I. since the beginning; they are very nice medals, and it seemed, she said, a pity that they had to be given up when retiring from the institute.

### Nursing in the Straits Settlements.

By a Hospital Matron.

All who are nursing at home in large, well-ordered hospitals, with everything ready at hand, and every latest convenience for patients' comfort and welfare, would open their eyes in wide amazement, and probably exclaim in horror at the way we in the colonies have sometimes to manage things.

I am in the Straits Settlements, in charge of a European hospital containing four beds. The building is an old bungalow, containing a dining-room and three bedrooms, with a verandah all round.

Some Drawbacks.

There is no proper entrance to my hospital, people being obliged to come in by the back way, or else up a broad flight of steps at one side, leading on to the verandah, and straight into the large bedroom already mentioned. Strangers usually come this latter way, and, not knowing any better, walk into the first door at the top of the steps, and so in by the bedroom. Now this is all very well when the room is empty, but when, as is often the case, it is occupied, it is a serious annoyance to the patient. It is all very well to say why not keep the doors closed and put a notice on? If that were done the patient would die of suffocation, as by shutting these doors you shut out most of the air. Another drawback to my hospital is that it is so close to the railway station and the main road that patients who require to be kept quiet are constantly annoyed by trains and their attendant noises, besides other traffic. I can even fancy nurses at home smiling at the thought of trains and traffic in the far East. Oh! but I assure you, we live in a centre of commerce, and this town is one of the principal ones in the Straits. We boast of a station lighted by electricity, where there is a constant rush of trains going out and coming in. True, there are only three directions in which these trains travel, but from the bustle and noise one might imagine oneself at St. Pancras. Of course, it is a great annoyance to a bad case in hospital, and we have at last succeeded in representing the bad state of affairs to the ruling Powers, with the result that we are to have a new European hospital built with all modern improvements far away from trains and other disturbing elements.

A Small Staff.

The nursing staff consists of one, and sometimes the demand on her energy is so great as to be almost impossible to meet it; other times the beds are empty and the staff has nothing to do. If all four beds are filled, which occasionally happens, with only one out of the four, say, needing night work, my hands are full day and night, with very littl time for rest. In such circumstances, where second help is absolutely necessary, I am obliged to call in a dresser from the general hospital for a few hours' relief. But the dressers are hopelessly careless. They will follow out written directions from hour to hour as long as you are careful that the directions are full and explicit in writing. Should you by any chance tell them to repeat three-hourly treatment, for instance, they will say, "Yes, they fully understand"; and as soon as you have departed they make the most glaring mistakes.

A Case of Enteric.

My first case was a Japanese planter with enteric fever. The patient was delirious, and of course demanded constant attention day and night. I remained on duty for nearly 48 hours, and then applied for relief. A dresser was sent me to relieve for meals, and from one p.m. till about eight p.m. So that I was on duty all night and till one p.m. next day. That seems long hours to anyone at home, and so I found it out here. I found also, to my dismay, that the dressers left in charge were absolutely untrustworthy, and had the vaguest ideas of disinfecting, so that as soon as I could I dispensed with them, and ask for their help now as little as possible.

Illness of the Staff.

Unfortunately for me, when my carefully-tended typhoid had reached the end of the third week the staff was attacked with fever and obliged to succumb. The beds were then all occupied, but there was only one other serious case besides the enteric. So the dressers were in demand once more. My coadjutor, who is engaged in private nursing, but has absolutely nothing to do with the Government work, was asked to go over occasionally and superintend. She did, and found, to her horror and mine, that my enteric case was sitting on the verandah in the heat of the day, and the dresser on duty asleep. This circumstance completed the cure of the staff, for she came on duty and resumed her sway. Luckily there was no relapse, and the patient recovered rapidly and went his way. A few months ago the staff got rheumatism so badly as to be unable to walk, and was ordered for change to a very dry spot some 40 miles from where she was nursing. The journey was a tedious one, and, being unable to move about, an ambulance had to be arranged by which she was taken from the train to the boat. As she was being raised on high by four dusky coolies she caught sight of her old enteric case looking the picture of rude health.

### Some Hospitals in the Netherlands-Indies.

### I.—BATAVIA: THE MALAY HOSPITAL.

By a Nurse on Her Holiday.

The point for which the ordinary globe-trotter, with but a modicum of time to spend over her rambles, usually makes when visiting Java from its western end is Buitenzorg. My hospital duties only allowed me four months' leave, and when I landed in Java I turned my attention to Buitenzorg accordingly. It is a picturesque but dull—and to English ideas somewhat over-rated—country town. It was established by the Dutch prior to our occupation of the island in the early part of the century now expiring, to afford relief to the easy-going and well-to-do officials and company wallahs of old Batavia—that erstwhile city of calentures and flux, and of commercial corruption too; and, we may now conclude, of anopheles galore, though Professor Koch, who had left it only two days before I stepped ashore there, tells us that except at Jakatra and Tandjong Priok the locality is only moderately stricken with that pest at the present day. But times have changed, and with them Batavia. The old city by the boom and the Kali besar still receives by day, it is true, its merchants, its bankers, its clerks, and its officials; but by eventide all these migrate (mostly by steam-tram, be it said) to the newer and well-laid out suburb of Weltevreden, and leave the littoral and more low-lying purlieus to a horde of Chinese, Malays, Bugis, Bantamese, Japs, and mixed bloods of every conceivable oriental blend, whose dwelling-places are as little European in style as they are in their unsavoury character and general surroundings.

### The Buildings and Patients.

There is an interesting hospital for natives in the midst of all this mal-sanitation, to which, by the obliging disposition of H.B.M.'s Consul, and of Colonel De Freytag, the Director-General of the Medical Department of the Netherlands-Indies, I was permitted access. It is situated near the Glodok Bridge, on the Jakatra side of the tram avenue, and is in charge of a Dutch resident medical superintendent, assisted by two Javanese practitioners, locally qualified after a careful training, with periodical examinations under Government auspices extending over eight years. The wards are constructed chiefly of brick and plaster, paved with black marble, and roofed with red tiles; but some of them are panelled with plaited strips of bamboo matting instead of brickwork, and afford quite enough protection against the mild and stagnant climate of Batavia. The buildings are arranged in pavilion fashion; but, owing to the scarcity of land in this central part of the busy city, they are distinctly huddled, and the ground — like all that part of the old town—is low-lying and damp. As regards patients, the hospital can accommodate between six and seven hundred, but contained only some two hundred or so at the time of my visit. Of these I was sorry to find no less than sixty-five occupied the female lock ward, being almost all Japs, with a sprinkling of Chinese, and here and there a Malay woman.

### The Wards and Furniture.

One ward was devoted to prisoners, for whose custody the hospital steward (an Indo-European) informed me he had to be responsible, although he was not definitely constituted a prison official. Another contained mostly malarial cases, many of them with big spleens that, on palpation, were easily felt through the abdominal wall, and seemed as large as a man's head and almost as hard. In another the accidents were collected; they were only eight, and none very serious; which is remarkable in a city of 180,000 inhabitants, where all the vehicles are driven by natives, and there are steam trams, electric trams, and canals in the streets into the bargain. No operative work of any magnitude is done here, it being sent on to the Military Hospital at Weltevreden; and the natives are generally strongly averse to surgical procedures being carried out upon them. The usual open conduit system of sewage disposal, in vogue all over Java, is here followed. It may have advantages; its drawbacks are more obvious. The bedsteads supplied to the native hospital are of iron, with teak-framed mattresses fitted with "cane-seated" work in place of slats, exactly like those in use in the hospitals for Europeans in Java, and all are made in the Colony.

### A Case of Leprosy.

I saw a case of nodular leprosy here, being treated in a general ward, where the poor fellow had been for four years. He was not in a very advanced stage, and when I asked one of the Javanese house physicians why they kept a patient of that kind so long in the hospital he said that he had got worse so slowly that they had some hopes of his ultimate recovery, an explanation which set me thinking, and I am not even yet sure whether the good man's logic was sound or otherwise. The local term for leprosy appears to be merely "Sakit besar," which is "the great sickness" in Malay. I also noticed a bad case of yaws—a disease of which I had seen much in the South Sea Islands—and learned that the Javanese name for it is "patek." It was doing well under two grammes of iodide of potassium daily, with a nitrogenous diet, including lentils, by way of a prophylactic against beri-beri. I saw a few cases of the last-named curious disease here, too, but shall have to refer more at length to that in describing the beri-beri hospital at Buitenzorg in a future paper.

### No Nursing.

There was virtually no nursing in the native hospital at Jakatra—that is, "nursing" as we understand it. The attendance was afforded by the Javanese house physicians, assisted by a few Malay servants. I came away from this hospital—the Ruma Sakit Malayu, or Malay Hospital, as it is commonly called—feeling that the Dutch have done well to establish such an institution, and that their liberality in admitting all patients gratuitously is indicative of the good intentions of the Government towards the native population, even the non-aboriginal portion. I had not the good fortune to meet the medical superintendent, as he was absent at the time of my visit; but the impression the place left in my mind was that, creditable as it is, I wanted something more to satisfy my ideal. But what nurse who has worked in a hospital for native patients, of whatsoever nationality, does not sigh for a closer approximation to her ideals? I am sure that your correspondent at Lagos, amongst others, will sympathise with me. And so I passed on my way to Buitenzorg.

### Death in Our Ranks.

Sudden Death of a Matron at Cape Town.—The sudden death is reported of Miss B. Walle, matron of the City Smallpox Hospital, Rentzkie's Farm. Miss Walle was the daughter of the late Rev. Henry Walle, professor of Oxford University, and went to the Cape some time ago with her sister, Mrs. Hill, who is also a nurse. She was appointed matron of the smallpox hospital last January, and was very popular. The *Cape Times* gives an account of the funeral, which was attended by several nurses, as well as by a large general congregation. The coffin was borne by four members of the sanitary staff in uniform.

The Late Miss Forrest.—There was a striking demonstration of sorrow at the funeral of Miss Frances Forrest, lady superintendent for nearly a quarter of a century of the Nottingham and Notts Nursing Association. During her *régime* the private nursing of the association was established on a sound and businesslike basis, and is now a financial success, with a staff of between thirty and forty trained nurses. Miss Forrest's great achievement was, however, the organisation of nursing amongst the sick poor, by whom she was well known and much respected. In other circles her quiet and unostentatious manner secured her many warm friends.

### Across the Seas.

### NURSING IN CEYLON.—THE LADY HAVELOCK HOSPITAL.

By a Correspondent.

Every nurse who touches at the port of Colombo wants to see this model little hospital, which was suggested and opened by Lady Havelock, wife of a former Governor of Ceylon. It is staffed entirely by women, and was built for the benefit of the native women whose religious customs and traditions forbid the ministrations of medical men.

The educated Cingalese woman does not object to being attended by a physician of the opposite sex. But Mohammedan and Cingalese women of the working class, till Lady Havelock came to the rescue, suffered seriously from the absence of skilled medical treatment.

The resident surgeon at the Lady Havelock is a qualified Dutch burgher—the first, and so far the only woman who has studied and taken her degree in Ceylon. A medical woman practising in Colombo is the senior visiting officer to the Havelock, which contains ten wards, built on the pavilion plan. The hospital stands in the centre of beautiful grounds, full of big, shady tropical trees and vivid flowers.

### The Patients.

Cingalese native women suffer from every kind of uterine trouble owing to the malpractices of native midwives, and the admirable little theatre is kept busy by the operations necessary to correct the sins, negligences, and ignorances of these unauthorised practitioners.

Six paying patients are received in the pretty private rooms reserved for such cases. Three rupees a day (six shillings) and ten rupees entrance fee covers the cost of nursing, medicines, medical attendance, and board.

Many private patients bring an ayah, or native attendant, and a European trained nurse—if they can get one. But trained nurses, unfortunately, are scarce in Ceylon.

### The Nursing Staff.

At the date of this visit the nursing staff consisted of an English matron and assistant matron, ten Dutch burgher pupils and nurses, and two Cingalese probationers. It is a very difficult matter to induce Cingalese women to undergo a hospital training. So far, no native gentlewomen have taken up nursing, though the English matrons affirm that the native woman, properly trained, would make an ideal sick nurse. A qualified woman dispenser resides in the hospital, and the only masculine faces seen about are those of the cooks and sweepers. An oral examination is held and a certificate granted to the nurses after one year's service in the hospital. As soon as they are certificated there are plenty of posts open to them in the out-station and up-country Government hospitals, where a year's certificate entitles them to an appointment as charge nurse or superintendent. All the pupil nurses are paid from the first. They come from a class which could not afford to give services in return for training, and the salary is ten rupees (£1) a month, with uniform, laundry, and shoes. Groups of the nurses were off duty and playing croquet on the lawns, the two Cingalese being initiated into the mysteries of the English game, and all looking very nice in their white-braided brown holland uniforms.

### Hours of Duty.

The nurses have nominally twelve hours on duty, but three hours of this are devoted to rest and meals, so that they have a nine-hour working day, which is quite long enough for the tropics, though there is no ward cleaning, scrubbing, or manual work to do. All these domestic duties are performed by "sweepers" and native servants.

The day nurses go off at six p.m., by which arrangement they get a pleasant opportunity of air and exercise in the cool of the evening. Night nurses go off at six a.m., so that they can take their recreation in the early morning hours, before the scorching sun drives everybody indoors.

The native women seem to be pleasant and tractable patients. Nightgowns are garments unknown to native Ceylon, and it is curious to see the patients in bed with lengths of linen rolled around them, these doing duty as bedgowns.

### The Friends of the Patients.

The grandmother, mother, and children are admitted with a patient if she wishes it. These family parties squat on the ward floors and indulge in unlimited curry, rice, and gossip. Such supernumerary patients are dressed in hospital clothes, and are not allowed outside the gates. A native gate porter keeps a keen watch over their movements, for, if permitted beyond the bounds, they bring back contraband spirits and betel nut. Betel chewing, with its subsequent expectoration, would prove an insanitary nuisance, and mar the delightful cleanliness of the floors. Both the patients and their friends are very fond of annexing ward thermometers, scissors, and surgical instruments. Anything bright and shiny appears to have an irresistible fascination for them. They display a wonderful faculty for concealing such treasure trove in the single cloth which forms the entire wardrobe of a Cingalese woman. Sometimes a hospital sheet, blanket, or other miscellaneous property is tucked beneath the native dress, so that the nurses have to look very sharply after surgical instruments and ward belongings generally.

### A Peculiar Regulation.

A curious official regulation demands that all the old linen of the Ceylon hospitals shall be burnt every three months. A Government official comes round on a periodic linen inspection, and condemns holey sheets, towels, and so forth to a fiery fate. He does not leave the hospital until every atom of the condemned linen is reduced to ashes. The idea the Government has in view is to prevent the new articles supplied from being stolen, and the old substituted in their stead. A less wasteful plan would be to cut discarded sheets into four or five pieces, and so to dismember condemned towels and pillow cases that there would be no possibility of using them in place of the new linen. Throughout Ceylon the nursing staffs bewail the destruction of linen which would prove so useful in dressing burns and wounds. To use lint for such purposes in lieu of old linen is an unnecessary extravagance. But it is an extravagance we commit very largely in the London hospitals.

### The Cooking.

The kitchen arrangements of the Lady Havelock would somewhat surprise an English hospital cook. Native cooks rise superior to "kitcheners" and patent stoves, and all they need are several smouldering blocks of wood and a few saucepans. Given these, the Cingalese will produce an admirably cooked hospital dietary of rice, butter, fish, and appetising curries. The performance is a culinary triumph.

Visitors to the hospitals of Ceylon can give only one verdict —that they are very much behind the times. English matrons and head nurses are appointed. But red tapeism and official regulations prevent the progress in nursing which ought to mark the end of this enlightened century. Some day a wave of nursing progress will pass over the island. Already more than one member of the Government talks of "nursing reform." Many of the hospitals are excellent so far as building and equipment go. All that is needed s raised standard of nursing and the establishment of two or three training schools.

### Notes on Rectal Nursing.

By a Trained Nurse.

### I.—HÆMORRHOIDS.

In private practice the nurse comes into contact with many cases of hæmorrhoids and rectal troubles complicating other diseases, and she is constantly called upon to nurse hæmorrhoidal operation cases. All rectal diseases are of so painful a nature that the nurse who can give relief in the many simple easy ways possible is regarded by her patient as a true friend in need. "Injections for women and all local applications should be three degrees hotter than for men" is the axiom of one of the leading rectal surgeons. The nurse who bears this in mind will please her patients of both sexes, for it is a rule that has no exception. The painfulness of piles has almost passed into a proverb, and the nurse who makes a speciality of rectal cases needs to cultivate a tender touch and a thoughtful consideration. I have known a patient with rectal ulcer and fissure to lose all confidence in his nurse when she gave him, on the eve of operation, an enema of strong soap and water. The additional two or three hours' smarting and severe pain caused by the use of the soap was as unnecessary as it was thoughtless. Oil and water or, better still, oil and barley water is the proper injection for bad hæmorrhoidal and ulcerative rectal conditions. The somewhat rough and ready methods of giving injections in ordinary surgical cases are quite unsuited for use in rectal troubles.

Many hæmorrhoidal patients are treated without operation; rest, injections, and lubricants forming the chief part of the treatment. Hæmorrhoids—more or less serious—will be found complicating liver and heart cases.

In all rectal troubles which are at all serious and painful it is a great relief to the patient to pass the motions lying down. If a tendency to prolapse exists, this position in itself frequently averts the prolapse. If the hæmorrhoids are liable to "come down" after a motion the recumbent position will often prevent this. Sometimes this position alone entirely stops the hæmorrhage from piles, which would bleed profusely if the motion were passed whilst sitting. Nurses should remember that in the sitting posture a large part of the weight and pressure of the upper half of the blood column is thrown on the rectum. In the horizontal position this pressure is not exerted, and in rectal troubles it is a great point gained to relieve the rectum of this weight at the time the motions are passed. It affords considerable relief to the acute suffering of a fissured and ulcerated rectum for the motions to pass while the patient is lying on the left side. The parts should be thoroughly bathed afterwards with a soft sponge and warm soft water (rain water should always be used when obtainable), and dried with a hot soft towel or a heated pad of absorbent cotton. Many of the toilet papers used in the sick room are totally unsuited to ordinary cases, but they are positively painful and harmful when there is any abrasion or an inflamed condition of the delicate mucous membrane of anus and sphincter.

Before the surgeon examines for hæmorrhoids a warm water injection of about a pint of barley water is usually given, and some surgeons prefer to moisten the examining finger with soap and water rather than with oil or ointment. In fissure and ulcerated cases vaseline or carbolic oil is used—the soap is apt to prove painful. For non-operative cases of hæmorrhoids cold water injections may be employed. The common method is to use a three-quarter pint injection of warm water to break up and bring away the motion, following this with a three to four-ounce injection of cold or iced water. The patient should retain this for five to seven minutes, or the surgeon may order it to be retained altogether and absorbed. This cold water treatment is used for its tonic and astringent effects where the case is too mild for operation or where the hæmorrhoids are a painful complication in advanced pregnancy, heart, or other cases unsuited to operation. If lubricants or ointments are prescribed locally, the small ointment bottles made specially for rectal cases are most convenient. These are small two-ounce lead bottles, similar to artists' colour tubes. The stopper is unscrewed, and a rectal nozzle with several small openings round the sides and at the tip is slipped on the tube. By squeezing the ointment tube the lubricant is distributed evenly and comfortably in the rectum.

The nurse must remember that the knee-chest position, which must, however, not be used in heart cases, gives great relief to painful and congested rectal conditions; and hæmorrhoidal inflammation is much relieved if the patient lies flat in bed with the pillow barely raising the head. Hot sponge fomentations wrung out fresh every ten minutes, applied to the anus, and kept in place by a flannel T bandage, give great relief in tenesmus, anal spasm, and cases of inflamed piles. In such cases some surgeons, however, will prefer the use of a rectal ice-bag. The ice should be pounded, and a bladder makes an excellent substitute for a bag. When severe rectal congestion and ulceration is present, blocks may be placed beneath the foot of the bed, so as to raise the pelvis and prevent venous congestion. A pillow may be placed beneath the hips for the same purpose. A half-sitting position in bed or piled-up pillows should not be allowed in any rectal cases or in dysentery or severe diarrhœas, as this position throws the weight and congestion of blood on the rectum, where it proves most harmful.

The utmost local cleanliness is necessary in every type and condition of rectal disease. Plenty of soap and soft water should be used locally. It is only as an internal remedy that soap causes pain. It should be freely used externally, and the drying should be done by means of heated towels or cotton pads. The least friction is harmful and painful. Where powder, boracic, iodoform, or a mixture of the two are ordered, the first application should always be carefully washed away before a fresh application is made. A good deal of local irritation may be caused by neglect of this simple rule.

In non-operative cases bougie treatment may be employed. A well oiled bougie is passed after every motion and retained for ten to fifteen minutes as a support and tonic to the rectum. Simple external piles are often treated by a lancet cut, the removal of the clot forming the pile, and the application of a hot linseed poultice.

More serious and complicated are internal hæmorrhoids, which cause a severe tax on strength and vitality by constant bleeding. A very slight hæmorrhage, from its daily occurrence, constitutes a serious drain on the constitution, and in addition the internal hæmorrhoids are apt to become inflamed, and are liable to protrusion as well as being the origin of much pain and discomfort.

An external hæmorrhoid is constituted by a distended condition of the veins outside the sphincter. An internal pile is when the veins—and sometimes the arteries too—of the mucous membrane within the sphincter are congested and enlarged. Internal hæmorrhoids sometimes protrude outside, but they are internal all the same. Constantly overloaded bowels, uterine displacements, and a slow or obstructed circulation, as in heart and liver cases, cause hæmorrhoids. Chronic constipation too gives rise to them, owing to the rectum being kept in a constant state of dilatation. The bleeding after a motion is caused by a slight rupture of one of the congested veins.

### A Modern Aseptic Operation.

By A Nurse in Attendance.

I was very much interested a short time ago in helping at a thoroughly up-to-date aseptic operation. After an absence of some time from the hospital world it was most instructive to note the change that had come over the spirit of things surgical—to realise that "the old order changeth, yielding place to new" in surgery, as in other matters.

The case was an abdominal section for double ovariotomy, and was done in a private house. The nurse who was with me in charge of the case had never before seen an aseptic operation, and was as much interested in it all as I was, though nurses are almost *de trop* on these occasions now-a-days—*de trop*, that is at the actual operation—their work is mainly done on the evening before, and hard enough work it was. The surgeon brought us most explicit written directions as to what he required, and what was to be done with every utensil to be used in the operating room; he had omitted no smallest detail.

On the evening before, then, the nurses' preparations began. All the basins, jugs, baths, &c., required were taken up into the bathroom, and there they underwent a most exhaustive cleansing. Scrubbing with soap and water began the process, then they were all "gone over" with methylated and then with turpentine, and finally they all had to spend the night in a zinc bath filled with a solution of lysol, this solution covering all the crockery. In this, too, were placed the surgeon's own mackintoshes, and the mackintosh coats of himself and his assistant, also the towels he was going to use on the morrow. And in this solution the articles all sat until the hour of operation.

On the afternoon preceding the day itself the surgeon came to the house and himself scrubbed the patient's abdomen thoroughly with soap and water, and shaved the almost invisible hairs from the surface of the skin, after which a large compress of folds of lint wrung out in a lysol solution was applied, and kept on until the moment of operation came. On the operation morning, and on the evening before, the nurse douched the patient with a solution of lysol.

For the actual operation the basins, &c., were disposed on tables in convenient positions in the usual fashion, two or three containing lysol solution for the surgeon's hands, for the needles, &c. Large jugs of the solution were also placed ready for use, and a kettle was on the fire. Before the operation began the surgeon and his assistant removed their coats and donned the dripping mackintoshes which had been reposing in lysol. Their arms were bare, and were also dipped in lysol, after having been exhaustively scrubbed with soap and water and a nail brush. The whole of the patient's abdomen, except the portion to be immediately operated upon, was swathed in towels wrung out of lysol solution.

During the operation the nurses had absolutely nothing to do but stand and watch, except for occasionally filling up the kettle and seeing that the surgeon's basins of lysol were kept fresh for him. No sponges to wash, no instruments to hand, no needles to thread. The nurse at an aseptic operation where an assistant is present is a drug in the market. The surgeon brought his own sponges, of sterilised wool and gauze wrapped up in lint, and the bundle of them was laid on the operating table upon a towel which had been wrung out in lysol. No one touched those sponges but the surgeon and his assistant. The instruments were on a table to the surgeon's right hand, and had been boiling almost up to the moment when they were wanted. The assistant manipulated artery forceps, and threaded needles; the nurses, as I say, did nothing till it came to the time of the dressings, when they were allowed to cut the strips of iodoform gauze, which the surgeon then wrung out in lysol solution and applied to the wound, surmounting it with a large pad of blue wool. That wound was a very beautiful sight when the first stitches were taken out. It had healed by first intention from top to bottom. It was a wound of between four and five inches in length, and it looked the very picture of a healthy, satisfactory scar.

### The Nurses' Bookshelf.

[We invite Correspondence, Criticism, Enquiries, and Notes on Books likely to interest Women and Nurses. Address, Editor, The Hospital (Nurses' Book World), 28 & 29, Southampton Street, Strand, London, W.C.]

Mona Maclean, Medical Student. By Graham Travers. (Publishers, William Blackwood and Sons. Fifteenth and Cheaper Edition).

It is not surprising that Dr. Margaret Todd, the authoress of "Mona Maclean," who at first concealed her personality under the pseudonym of "Graham Travers," has achieved a remarkable measure of popularity by her essay into the realms of fiction. Her novel has many solid merits, and is written so brightly and vigorously that no one who takes it up can fail to read it to the end. One of its charms is undoubtedly the fact that the writer has herself had the professional experience which she depicts so cleverly, while another and still greater is the strength and sweetness with which she invests the character of the heroine. Although it would not be fair to say that the interest entirely centres in Mona, her movements, her self-denial, her buoyancy, her tenderness, and her unconsciousness of her own attractions, absorb so much attention that there is a risk of the other personages in the book being too lightly esteemed. The masterful doctor, the old clergyman, the shop-keeping cousin, the aristocratic baronet and his family, are all careful studies. It is a striking proof of the ability of the authoress that though the prejudice against women workers is fast subsiding, and the book was written some years ago, her treatment of the subject is still fresh and to the point.

"Before Good Night" and "From Door to Door." One Volume. By G. H. Dabbs, M.D. (Published by C. Deacon and Co. London. 2s. 6d.)

These stories, although written on the author's part with sincerity, fail, in spite of it, to be convincing. There is an effort at spontaneity, but it lacks reality; and the effusive sentimentality of the style, attractive, perhaps, to some readers, would be unintelligible to a child, for whom they are ostensibly written. The elements of romance are found in the friendship of a medical student for a little girl flower-seller whom he finds asleep under a railway arch. He befriends her and her crippled boy-chum, gifted with a beautiful voice, who lives with an old soldier grandfather. Nina he sends late into the country to be educated at the village school, under his mother's care. After an interim of hospital nursing she becomes his wife. There is some good writing in the book, and those readers who appreciate the moral sentimental school of writing will enjoy the perusal of this well-got-up volume, which, if its literary merits equalled its author's good intentions, would be above criticism.

The charming dedication on the opening page must be quoted:—

"A Toi Nina Camarade.

"And now I give you to keep for ever and ever
The story of days and of nights that centred in you,
The fable a fairy once wove by the light of your eyes!
For 'love of your love' was the bud of the flower of endeavour,
And desire of your praise the only incentive I knew,
And the 'silence of tears' your sweetness of shy surprise."

There is much stray verse scattered through these pages, of which the lines given—an evening hymn, ending the first story, and a wedding canticle, are the best specimens.

### Echoes from the Outside World.

### AN OPEN LETTER TO A HOSPITAL NURSE.

Everyone who knows Lord Roberts will understand the pain it caused him to confirm the sentence of death passed upon Hans Cordua by the Court for his part in the conspiracy to kidnap the Commander-in-Chief and to murder his officers. But even the stoutest opponents of capital punishment will, I think, hardly care to deny that in this case it was absolutely essential to take the life of the criminal. It is due to him to say he died without any manifestation of fear. If Cordua had not been shot, others would have been confirmed in the belief that, whatever outrages they committed, they were secure, in the event of detection, from the awful penalty of an ignominious death. The Boers, who will not fight on the square, and who resort to treachery, to lies, and to every poisoned weapon they can use, now know that kind-hearted and merciful as Lord Roberts is, he will not hesitate to employ the most severe measures if they are proved to be necessary. He has been "slow to anger" in the best sense throughout the war, and has consequently incurred the adverse criticism of people at the Cape and at home who possess neither patience nor wisdom. But he has shown, at the right moment, that there is a limit to his endurance, and the enemy in South Africa cannot fail to realise that any acts contrary to civilised warfare will, in future, expose the authors to certain and adequate retribution. So long as there are 20,000 Boers, or anything like that number, in rebellion, the conclusion of peace remains, of course, out of the question. The capture of General Olivier and his three sons will tend in that direction.

It is all very well for us to offer hospitality to native chiefs from India, but the Viceroy, with much courage, has issued a circular in which he says that their repeated absences are regarded as a grave dereliction of duty. In fact, Lord Curzon takes such a serious view of the matter that in future leave for travelling will only be given "where it will result in personal and public advantage." Both in England, and even more in Ireland, we know something of the evil consequences due to the absenteeism of landowners, and it is easy to understand that the constant trips made by certain princes to Europe, notably by the Gaikwar of Baroda and the Maharajah of Kapurthala, have had a bad effect. The trips are not merely constant, but costly, and, while they diminish the influence of the native chiefs among their own people, they also tend to the impoverishment of their own estates.

All nurses who are cyclists, and their name is legion, will be glad to hear of the action of Admiral Field the other day with regard to three publicans at Porchester who had refused to supply two lady cyclists with tea. It does not appear whether the reason for the refusal was that the ladies wore rational dress or whether the landlords objected *in toto* to women cycling, but the fact remains that they had asked for tea and been refused, probably in the hope that they would fall back upon some drink of another character. The ladies, with much public spirit, for which the gallant Admiral thanked them, as all lovers of fair play must do, summoned the publicans, and the decision was given that if tea was demanded, tea must be forthcoming, as by their licence they were bound to serve anyone calling at their houses with reasonable refreshment when civilly demanded. The sting of the admiral's remarks was, like that of the wasps, in the tail. He wished "the trade to know that the law would be upheld." A good example always unconsciously impresses others, and many magistrates will, without doubt, follow in the steps of Admiral Field. This will mean far greater comfort for women cyclists, and probably also for male tea drinkers. It has often happened hitherto that a woman has gone tea-less on her way unless a hospitable cottager could be found, rather than risk a curt refusal at a suburban or village inn. The better class of publican has for some time realised that it answers his purpose to cater for persons of both sexes who do not necessarily require alcohol as a portion of their refreshment.

I wonder how many of you know Budleigh Salterton? Very few, I expect, because—although it has a railway of its own from Sidmouth Junction, and is less than 200 miles from Waterloo on the London and South Western Railway, a journey taking about four hours—the little watering-place is content to go on its own way without obtruding itself, and only those who happen to have friends there ever hear what a pretty spot it is. As you emerge from the station there are fields, green and golden, up hill and down dale, with here and there clusters of trees, all lending beauty to the landscape. The place itself is hidden from view, but a few new houses with nice gardens show that you are approaching the haunts of men. In one of these villas I caught a glimpse of the son of a well-known actor, with his wife and child, who had found his way down to this lovely corner of Devonshire. Then we went along the winding road till we entered the principal street. On one side are the shops, on the other runs a sparkling stream, which flows merrily on its way, bubbling and tumbling over the stones. Beside the stream a great many of the houses are built, and as the distance across is too much to be easily stepped, there are diminutive—I had almost written "toy"—bridges, one for each house, if a large one, or to serve a few cottages with more humble requirements. So the little town stretches down to the sea, where there are some tolerably large private houses standing in their own gardens, called "Marine Parade," in which apartments can be had, and a small asphalted sea-front, with a shelter and a band-stand.

The weather had been rough the day before we visited "Salterton"—as they call the place here to save trouble, and also to distinguish it from East Budleigh, which is inland—and it was a glorious sea: great roaring waves coming in with a burst and a bound, dashing in clouds of white, feathery spray against the dark red sandstone cliffs, and the shingle beneath. The air was fresh and invigorating, and though there are many visitors, everything was so quiet and unconventional that I came to the determination that for real rest and change nurses could not do better than repair to Budleigh Salterton. The catering and shopping would be easy enough, as the "stores," as the Americans call them look good, but unfortunately it was early closing day that particular Thursday. We were very sad that we could buy nothing (I always thought that seaside resorts gave up their holiday afternoon during the season), for after living in a farmhouse a mile or two from anywhere the sight of an emporium developsa sudden love of spending.

Though rural, Budleigh Salterton has its entertainments. On Saturday a croquet tournament caused great excitement. If croquet is not, as a rule, quite so popular this year as last, there are exceptions, and at Budleigh Salterton the number of entries constituted a record. Moreover, the attendance each day of the play was much above the average. Lady Clayton was one of the competitors, but Mrs. Thomson, a local lady, won the challenge cup, and defeated the clerical holder of it last year.

### Typical Patients.

### THE HEN-PECKED HUSBAND.

A CONVALESCENT home affords a field for observation of types of character even more, perhaps, than a hospital, inasmuch as the patients are less under restraint, and in a more normal condition physically and mentally. I write from such an institution, situated in one of the Midland counties, twelve miles from a large manufacturing city, whence the majority of the patients come. These, chiefly of the artisan class, meet here upon a footing of equality, and are subject to the same conditions, but character is quickly manifested in widely divergent aspects, *e.g.*, the arrogant by nature, and the humble, those who are born to rule either by force of character or domineering instinct, and those who seem equally born to submit through indolence or weakness. An example of the latter kind, in the person of a hen-pecked husband came before me recently. Scene: Superintendent's office. Present, superintendent seated at desk. Enter a little man, nervous and deprecating in manner. Accompanying him, and obviously keeping him in tow, a little woman, who is self-assertion from head to foot, his "missis" in every sense of the word. She wears a hat crowned with nodding feathers, which oscillate violently as she emphasises her speech with tight nods and shakes of the head. "I'm agoin' to take him home. If he can eat he can work, and he's no call to be wasting his time here, a-tearin' about all over the country." "I hav'nt bin tearin' about," pleads the victim in feeble self-defence. "You hold your noise—I'm about wore out" (her tongue goes like the clapper of a bell). "All the children is at home with colds, and the baby just turned a year old," . . . . "But surely," I interpose pacifically, "you don't want your husband at home to mind the children?" "That's my business. You'll excuse me, Miss, but I know what I want him for, and that's enough. I've married him, and I'll do what I like with him. I s'pose" (with crushing hauteur) "I can do what I like with my own husband!" Here, I reflect, is the "new woman" with a vengeance. She claims more than equality. I turn to the man. "Of course, you must do as you please; I have no power to detain you. You can go home with your wife, but it would be foolish to do so. You are not fit for work yet." "Yes!" interrupts the little woman, "you can please yourself, of course, but if you mean to come back at all you'd better do it now. If you don't come out o' this now, I go back without you, an' I'll bring the children an' leave 'em here, an' I'll go off on my own hook. I can always earn my living, thank goodness!" "I think I'd better go, Miss," says the poor man faintly; "I'm much obliged to you, I'm sure, for all your kindness."

I watch them depart down the drive, the man with a crushed air, carrying his bag, and the little woman, still nodding her head at intervals and apparently expressing her sentiments vigorously. The pair are followed at a distance by a group of interested spectators, men and boys, among them the small boy (that *enfant terrible*) who caused all the mischief.

On investigation I found that each time the little woman came to see her husband she asked him if he had been out, and he told her "Not beyond the grounds." He explained to the other men, "You see, hers so jealous. You should ha' heard how she carried on when she found out there was women took here as well as men. And I'm sure I never carries on wi' no women. One's as much as I can do wi'. I'm only glad to be at peace a bit, and I ain't a chap as the girls is likely to run after."

On this afternoon the little woman asked the same question and received the usual answer, when the small boy struck in with, "Why you 'ave, mister. You went to Bromsgrove wi' us this morning!" And "that was the way the quarrel began!"

### Appointments.

ROYAL EDINBURGH HOSPITAL FOR SICK CHILDREN.—Miss Evelyn A. Mansel has been appointed Assistant Matron. She was trained at Charing Cross and Guy's Hospitals, and has been sister at the Women's Hospital, Soho Square, matron at St. Monica's Hospital, Easingwold, and matron at the Cottage Hospital, Ashby-de-la-Zouch.

CANTERBURY WORKHOUSE INFIRMARY.—Miss Florence Kite has been appointed Superintendent Nurse. She was trained at Greenwich Union Infirmary, and subsequently she has been staff nurse at Camberwell Infirmary and assistant nurse at Eastory Infirmary.

THE INFIRMARY, SELBY OAK.—Miss Rose Nash has been appointed Superintendent of Night Nurses. She was trained at University College Hospital for three years, and has since been superintendent nurse at Highworth and Swindon Union.

TODMORDEN UNION INFIRMARY.—Miss Annie Banks has been appointed Nurse in Charge. She was trained at the Birmingham Infirmary, and has since been charge nurse at Barnhill Poorhouse, Glasgow. Lately she has been engaged in private nursing.

### Minor Appointments.

COOK'S TERRACE INFIRMARY, PANCRAS ROAD.—Miss Thomson and Miss M. Rodgers have been appointed Ward Sisters. Miss Thomson was trained at St. Saviour's Infirmary, East Dulwich, and Miss Rodgers at the Birmingham Infirmary. Since then they have both held the posts of ward sisters at the Poplar and Stepney Sick Asylum, and since December last have been on the staff of the British Hospital, Algiers.

RUCHILL HOSPITAL, GLASGOW.—Miss Kate H. Todd has been appointed Sister. She was trained at the Royal Infirmary, Bristol, after which she has been private nursing in London, being for a year attached to the private nursing staff of Fitzroy House, W. Since October last she has been charge nurse of the male enteric ward at the Park Hospital, Lewisham, S.E.

KENSINGTON AND CHELSEA DISTRICT SCHOOL INFECTIOUS DISEASES INFIRMARY, BANSTEAD.—Miss Mary Wallis has been appointed Nurse in Charge. She has previously been assistant nurse at the South-Eastern Fever Hospital; nurse at the Western Hospital of the Metropolitan Asylums Board; at the Holborn and Islington Infirmaries; and charge nurse of the sick wards at St. Pancras Workhouse.

TEWKESBURY HOSPITAL.—Miss G. Williams has been appointed Staff Nurse. She was trained at the Guest Hospital, Dudley.

NEWTON ABBOT UNION.—Miss Mary Fraser has been appointed Nurse. She was trained by the Meath Workhouse Nursing Association at the General Infirmary, Worcester.

### Adelaide District Nurses.

LORD TENNYSON has continued to manifest in South Australia the interest he evinced in nursing at home. Presiding at the annual meeting of the Adelaide District Trained Nursing Society, he gave an address, in which he eulogised English country district nursing, and made a strong appeal to his hearers to extend the operations of the Colonial organisation. The society has only been established six years, but its progress is unmistakable. During the past year nine nurses attended upwards of 2,000 patients, and paid 34,362 visits. They are in constant request among the poor in and around the city, and we note, with pleasure, that no complaint was forthcoming at the meeting of inadequate financial support.

### Everybody's Opinion.

[Correspondence on all subjects is invited, but we cannot in any way be responsible for the opinions expressed by our correspondents. No communication can be entertained if the name and address of the correspondent is not given, as a guarantee of good faith but not necessarily for publication, or unless one side of the paper only is written on.]

### NURSES' READING SOCIETY.

MISS MOBERLY writes: I should be glad if all members of the above society who joined it in July, 1899, would either send me their subscriptions for the year 1900-1 or would communicate with me if they wish to leave the society. Only nine members have, so far, paid the subscriptions now due. Twenty-five members who joined in July, 1899, have neither sent their new subscription nor let me know of any intention of withdrawing from the society. I shall not be able to send examination questions in September to any members who have not paid their subscriptions early in the month.

### NURSING IN AMERICA.

ALICE FITZGERALD writes: May I also be allowed to enter a protest against "An American Graduate's" statement *re* doctors and their supposed want of courtesy to nurses? I wrote last week, but fearing the letter was too stormy for your well-conducted journal refrained from sending it. "An Advocate for Tolerance" has, however, put the matter straight from her own point of view, and no doubt we all heartily endorse what she says. It must seem a little strange to some of us to hear a trained nurse—one who has devoted her life to the care of the sick and suffering—giving vent to such uncharitable sentiments as "An American Graduate" expresses under the heading of "Influenza." She says: "I can only conclude it was a most fortunate thing for all concerned that 'An English Nurse' did fall a victim to influenza, and so decided to leave the hospital and return to her English home." It is sincerely to be hoped that no English nurse would feel justified in saying such a thing—even of her bitterest foe! May I add that if "The Rejoinder" had been penned in a less bitter spirit it might have done good and been rather more favourably received.

"AN IRISH NURSE" writes: I have read with great interest "An American Graduate's" reply to "An English Nurse," and I agree with her in all she writes. America is a long way before the British Isles in nursing matters. Anyone who knows anything about America will agree to that. "An Advocate of Tolerance" is right if she is speaking about nursing in Ireland, where nurses are treated with the greatest kindness, but it is not so in England. Some doctors behave very differently, and the people in whose house they are nursing look upon "the nurse" as a sort of upper servant. Several English nurses have joined my institution in Ireland, and say they would not nurse again in England—the treatment is so different. I am at present with a "chronic case," a very sad one, which needs all the sympathy and help a nurse can give. Chronic cases may be monotonous and uninteresting, but shall we not get our reward "by and by," just as well as those nurses who have gone to Africa and elsewhere and laid down their lives in doing their duty? Indeed, our skill is not wasted if we can even in a very little help our patients to bear their terrible sufferings, though it is a fact that nurses refuse a "chronic case," thinking their skill would be wasted.

### PILFERING TRUNKS.

"NURSE D. G." writes: At the end of January this year I was leaving a hospital in the south-east of London, and thinking to save expense and trouble, I sent on my two trunks and bicycle by the carriers. It was Saturday morning when they left the hospital, and as they were only going to Frances Street I expected them to have arrived when I did, about half-past six p.m. My bicycle had arrived, I found, but rather the worse for the journey, as a spring of the saddle was broken, besides being scratched. The next day being Sunday I found I could not do anything to regain my lost baggage, but on Monday morning they turned up. Both locks were undone, and the things inside were disgracefully tossed about. At a glance I saw that my trunks had been ransacked, and at once lifted the tray from the cabin trunk to find my cash box. I was horrified to find it literally burst open and twisted into all shapes. My precious gold watch, three gold chains, and valuable trinkets and purse were all gone. After a great deal of trouble and inconvenience I sent in my claim to the carriers, and being able to prove to them that my trunks were pilfered in transit, I was rewarded after a considerable time with a cheque amounting to half the amount I had claimed. Nurse G. and I have had a cruel lesson in packing our treasures in our trunks and trusting them to others. I hope no others will follow our foolish examples.

### AN EXPLANATION REQUIRED.

"PATER" writes: Five months ago my daughter entered one of the principal London hospitals as a probationer. Since then she has passed the doctor and been successful at a written examination. Rule 2 of this institution enacts that "Before an applicant can be accepted for three years' training she must serve for a probationary period not exceeding three months in the wards," and yet at the end of five months her papers were still unsigned, and she was unable to obtain any assurance from the matron that they would be. I think such a breach of faith cannot be sufficiently exposed.

### PREMIUM PROBATIONERS.

"A NEW PRO." writes: I have always been much interested in reading the varied experiences contributed by nurses to your columns, and I think possibly intending pro. readers would like to hear my first impressions and short experience of three months' hospital life. I entered one of the four largest hospitals in London as premium probationer, fully determined to be prepared for anything, with plenty of hard work. I had committed to memory those splendid "Dont's" which you printed some time ago as hints to new probationers, every one of which I found useful, especially, "Don't know anything." I advise intending pros. to read THE HOSPITAL carefully every week, study elementary physiology and anatomy, attend St. John Ambulance classes, and pass, but when in the hospital tell it not in Gath nor breathe a word even to your favourite nurse, or you will be annihilated on the spot. The knowledge thus gained will be of inestimable value as a help to understand more quickly. The first ten days were the most miserable I ever spent in my life. In the surgical ward, where I was placed, there were two staff nurses and a regular pro., under, of course, a sister. Not one of these would show or tell me anything, and on my asking several times, "Is this the hospital way of doing things?" I got the reply, "Have no time to tell or show you. You must find out." Naturally, I did many things wrong, but time was found to lecture me unmercifully. However, the patients were most kind in giving me hints regarding my new duties. I also recollect that on the second day a kindhearted ward-maid of eight years' experience cheered me by saying, "Don't worry, nurse, and try not to care. It's a way they have in all the wards here of treating the premiums. Try and get used to it." I did, but my advice to others is, "Don't enter as premium if you can avoid it." From time immemorial it seems to have been the custom to sneer and dislike the premium probationers as a body, and surely we are not all bad. I have often inquired the reason for this general dislike, but the only answer I obtained was that although the duties are on a par with those of the regulars, ill-feeling is caused on account of us having off-duty at exactly the same time every day, instead of alternate duty or longer time. The last of my three months there a new sister was appointed. She was kindness itself in explaining or answering any question I asked in her very few spare moments. Rumour says that she was once a "premium." In spite of difficulties I like nursing and all my patients very much, and when I am attending to their wants I feel quite happy. I may, in conclusion, give three words of warning to intending pro's. Don't in an uncertain light address a sister as a pro. I did. Result—for the next fortnight I called everybody "sister" as a safeguard. Don't ever smile when helping a dresser, whatever amusing incident happens. I did. Result—sent at once on an errand as far off as possible. Reserve all smiles for the ward kitchen. Don't sit down in the ward until you know every doctor in the hospital. I did. Result—thirty minutes' lecture on hospital politeness.

### For Reading to the Sick.

The chief pang of most trials is not so much the actual suffering itself as our own spirit of resistance to it.—*N. Grou.*

Watch with me, Jesus, in my loneliness,
Though others say me nay, yet say Thou yes;
Though others pass me by, stop Thou to bless.
—*C. Rossetti.*

I toiled; my tools were taken from my hand.
I sought for more, and straightway was laid down.
"What shall I do?" I sobbed. Then saw I stand
O'er me my Master; and without frown
Thus did He pitying answer me: "Be still;
This is thy time to bear and Mine to do,
To thee and in thee, all My holy Will.
What I do to-day thou canst not know;
But thou shalt know hereafter," saith my Lord.
"On thee, and not by thee, must My work be wrought."
And thereupon some echoes of the word
That with a keenly hearkening ear I caught,
After hard struggles, brought me peace at length,
"In greatness and confidence shall be thy strength."
—*S. H. Palfrey.*

**Reading.**

Strive to realise a state of inward happiness, independent of circumstances.—*J. P. Greaves.*

You cannot at present change your surroundings. Whatever kind of life you are to live must be lived amid precisely the experiences in which you are now moving. Here you must win your victories or suffer your defeats. No restlessness or discontent can change your lot. Others may have other circumstances surrounding them, but here are yours. You had better make up your mind to accept what you cannot alter. You can live a beautiful life in the midst of your present circumstances.—*J. R. Miller.*

Our veiled and terrible guest (trouble) brings for us, if we will accept it, the boon of fortitude, patience, self-control, wisdom, sympathy, faith. If we reject that, then we find in our hands the other gift—cowardice, weakness, isolation, despair. If your trouble seems to have in it no other possibility of good, at least set yourself to bear it like a man. Let none of its weight come on other shoulders. Try to carry it so that no one shall even see it. Though your heart be sad within, let cheer go out from you to others. Meet them with a kindly presence, considerate words, helpful acts. —*G. S. Merriam.*

The soul loses command of itself when it is impatient. Whereas, when it submits without a murmur, it possesses itself in peace, and possesses God. To be impatient is to desire what we have not, or not to desire what we have. When we acquiesce in an evil it is no longer such. Why make a real calamity of it by resistance? Peace does not dwell in outward things, but within the soul. We may preserve it in the midst of the bitterest pain, if our will remains firm and submissive. Peace in this life springs from acquiescence even in disagreeable things, not in an exemption from bearing them.—*Fénelon.*

### To Nurses.

We invite contributions from any of our readers, and shall be glad to pay for "Notes on News from the Nursing World," or for articles describing nursing experiences, or dealing with any nursing question from an original point of view. The minimum payment for contributions is 5s., but we welcome interesting contributions of a column, or a page, in length. It may be added that notices of entertainments, presentations, and deaths are not paid for, but, of course, we are always glad to receive them. All rejected manuscripts are returned in due course, and all payments for manuscripts used are made as early as possible at the beginning of each quarter.

### Notes and Queries.

**The Editor is always willing to answer in this column, without any fee, all reasonable questions, as soon as possible.**
**But the following rules must be carefully observed:—**
**1. Every communication must be accompanied by the name and address of the writer.**
**2. The question must always bear upon nursing, directly or indirectly.**
**If an answer is required by letter a fee of half-a-crown must be enclosed with the note containing the inquiry.**

*Structurally Remodelled.*

(225) I should be much obliged if you would kindly tell me which are the hospitals (*a*) in London, (*b*) in the provinces, which have been most recently structurally remodelled and brought up to the latest standard of efficiency?—*X. Y. Z.*

There is constant improvement in nearly all the London hospitals. The London, St. George's, and Middlesex are now in the architect's hands. The cancer wards at Middlesex, recently completed, are up to date in every respect. The Evelina Hospital has been entirely rebuilt, and considerable improvements have been effected at Queen Charlotte's, as well as many others. As to provincial hospitals, full particulars may be obtained from "Burdett's Hospitals and Charities for 1900."

*Enteric.*

(226) What is the percentage of deaths from enteric in the fever hospitals of the Metropolitan Asylums Board?—*Matron.*

According to the last report on which we can lay our hands the mortality of all cases of enteric fever treated in the hospitals of the Metropolitan Asylums Board was 16·4 per cent. This fatality, however, varied very greatly according to the age of the patients, it being only 2·9 per cent. for those under five years of age, while for those over 50 it amounted to 43·7 per cent.

*Peeling.*

(227) I should like to know if patients ever "peel" after influenza, exactly in the same manner as after an attack of scarlet fever.—*Ilec.*

Hardly "exactly in the same manner," but still, they do peel sometimes. In fact, almost any pyrexial disease which causes profuse perspiration will occasionally peel. This we see sometimes after typhoid fever or rheumatism. Again, almost any erythema will in some cases lead to death of the superficial layers of the epidermis, and thus to desquamation, but although in such cases the desquamation may be similar to what occurs when post-scarletinal desquamation is badly marked, there is no disease in which such complete peeling in large sheets may be expected to take place as occurs in scarlet fever.

*Rheumatism.*

(228) Will you kindly tell me if there is a book published on the special treatment of rheumatism in its various forms, and the diet prescribed? What is the usual diet in the chronic form?—*Acid.*

Many special books have been published on the various special forms of treatment which have from time to time been employed in dealing with rheumatism. It must be confessed, however, that the diet prescribed depends much on the physician who prescribes it, and we fear that "Acid" will not readily discover any fixed rules of universal applicability in the management of that congeries of ailments which are apt to be included under the title chronic rheumatism, many of which are of gouty origin.

*Employment.*

(229) I am 65 years of age and have a comfortable home near the sea. I have spent many years (the last twelve in Germany) in teaching and nursing children. Although in good health I am not fitted now to be in a situation, but I could be useful to the aged or blind, or to delicate children. Is there any society which would send me them here, and is it any use to advertise in The Hospital?—*Mary A. H.*

An advertisement in The Hospital might possibly help. You could also write to the Secretary of the Society for Promoting the Welfare of the Feeble-Minded, 43, Victoria Street, Westminster, and ask if that society could make use of your home and services.

**ANSWERS.**

*Photograph of Miss Nightingale.*

(191) Mr. James Rowley, of 52, Fielding Road, Bedford Park, Chiswick, W., writes that he possesses a crayon enlargement of Miss Florence Nightingale which was drawn by himself from a photographic group taken in 1886 by his sister, who is one of the Nightingale nurses. It may be seen at any time at his address.

*The Hot-Air Treatment.*

(206) The address of Mr. Greville is 31, Upper Berkeley Street, Portman Square, instead of New Bond Street, as given in a recent issue.

**Standard Books of Reference.**

"The Nursing Profession: How and Where to Train." 2s. net; post free, 2s. 4d.
"The Nurses' Dictionary of Medical Terms." 2s.
"Burdett's Series of Nursing Text-Books." 1s. each.
"A Handbook for Nurses." (Illustrated.) 5s.
"Nursing: Its Theory and Practice." New Edition. 3s. 6d.
"Helps in Sickness and to Health." Fifteenth Thousand. 5s.
"The Physiological Feeding of Infants." 1s.
"The Physiological Nursery Chart." 1s.

All these are published by The Scientific Press, Ltd., and may be obtained through any bookseller or direct from the publishers, 28 & 29, Southampton Street, London, W.C.

### Travel Notes.

### LVI.—KNOKKE IN BELGIUM.

Do you want to enjoy a superlatively cheap holiday by the sea? If so, I can proudly declare the impossibility of bettering Knokke. You must not mind quiet and seclusion, for undoubtedly it possesses both—no shops, no bands, no casino, no kurhaus, no *nothing* as regards the gay world, but splendid air blowing both from the sea and downs, peace and rest, an immunity from the necessity of dressing three times a day, and a facility for making plenty of excursions.

### The Expenses of the Journey.

By judicious management these may be rendered almost infinitesimal. Instead of reaching Ostend (the nearest seaport) by the usual Dover route, take the Steam Navigation Company's boat from Tower Bridge, first-class return 10s. 6d. For Knokke you must take train to Bruges, and from thence a steam tram, cost just about 3s., a trifle more with luggage. Thus, you see, your entire journey, return included, will be under £1. Where could you go in England by the sea, first-class return, for £1? Nowhere, unless it might be to the cockney joys of Southend, if, indeed, you can honestly consider Southend a seaside resort, where you are hardly free from the mouth of the Thames. Be that as it may, it is certainly very different to the exhilarating winds that blow across Knokke from the North Sea.

### Living in Knokke Itself.

This may be done for £1 a week, or even less; to those intending to go there I shall be pleased to give all information. The modest hotels are not luxurious, but they are clean, comfortable, and the food excellent. I greatly deprecate people wearing out their old clothes when on the Continent, thereby causing us to be considered the worst-dressed nation in the world; but carefully avoiding that pitfall it is pleasant to feel that one may with a safe conscience wear the same costume all day without hearing the lamentable fact commented on by supercilious neighbours. It would be quite out of place to dress smartly for dinner, and in a good, well-fitting, tailor-made tweed, one feels properly attired for all occasions.

### Amusements.

Truth compels me to say there are none. If you want such do not go to Knokke; it is only suitable to those who are happy in a quiet, retired place, with such modest distractions as consist in visiting some of the old world Flemish cities of Bruges, Ghent, Ypres, &c. . . . which are all to be seen in a day's excursions. Those intent upon wild excitement can even cross the frontier into Holland and visit Middelburg, returning in the evening with the flushed triumph of successful explorers. You can visit Ostend easily and frequently, and mingle in the gay throng always to be found there, but to stay at Ostend does not suit slender purses; it is one of the most expensive places on the Continent. Blankenberghe, not so gay nor so expensive, is also within reach. The bathing is excellent and cheap, and, I believe, considered very safe. Between the village and the sea lie undulating downs, something resembling those of Sussex, and charming wild flowers peep up everywhere. The air is like champagne, and appears to me far preferable to that at Ostend. I think these open downs must be the cause of it, with their occasional whiff of scent from the modest little flowers that stud the short grass.

### Excursions Round Knokke.

There is a charm in feeling that one can get into Holland at a bound, a sense of vastness and freedom very agreeable to the toil-worn Londoner. Middelburg is a typical Dutch town, and well worth a visit; if you are cyclists you will wheel to Breskens, if not the train takes you there for a trifle. From thence you cross to Flushing, or Vlissingen, as it is called in Dutch, in a steamer which makes the transit six times daily; then train again to Middelburg, distant five miles.

There is a good deal to be seen in Middelburg, and I wish I had more space in which to describe its interests to you. There are two fine churches. The Abdy (Abbey), built in the twelfth century, but burnt in the fifteenth and rebuilt in the sixteenth—it is no longer used as a church, but by the Provincial Council, and contains some magnificent tapestry—and the Nieuwe Kirk, where Jan and Cornelis Evertsen are buried, two heroes who died fighting in the Dutch Navy against us in 1666.

Of the museum, with its extraordinary name, unpronounceable by English tongues—Zeevwsch Genootschap der Wetens chappen—I think nothing. I abhor and detest local museums, and never think them worth the entrance money; but in the old Town Hall there are some objects of interest, such as portraits of the Evertsens and the curious wooden seats originally used by the magistrates. There is a steam tram from Flushing to Middelburg, so that if hours are vexations, and one mode of locomotion does not suit, the other will.

### Veere, Goes, and Bergen-op-Zoom.

These three places are all easily visited, Veere by continuing in the train beyond Middelburg another three miles. It is quite a small place, but is on the whole more interesting than its more important neighbour, because it has preserved a good many of its old houses, and its town hall is very striking. Goes, on the rail between Middelburg and Bergen-op-Zoom, is interesting too; be sure to visit the Aosteinde Inn, once the castle of the Countess Jacqueline of Bavaria. Goes is the capital of the Island of Beveland. Before reaching it you pass the enormous Wilhelmina Polder. A polder is really land reclaimed from being an immense morass, or sometimes a lake. It is done by a wonderful system of draining in which the Dutch are past masters. The land thus reclaimed is extraordinarily fertile. Bergen-op-Zoom, distant an hour and a half from Middelburg, was once among the strongest fortresses of Holland, but is now dismantled. The church is interesting, and contains many monuments of note. Be sure to visit the town hall, where you will see one of the splendid chimney pieces peculiar to Holland and Belgium.

---

### TRAVEL NOTES AND QUERIES.

Rules in Regard to Correspondence for this Section.—All questioners must use a pseudonym for publication, but the communication must also bear the writer's own name and address as well, which will be regarded as confidential. All such communications to be addressed "Travel Editor, 'Nursing Mirror,' 28, Southampton Street, Strand." No charge will be made for inserting and answering questions in the inquiry column, and all will be answered in rotation as space permits. If an answer by letter is required, a stamped and addressed envelope must be enclosed, together with 2s. 6d., which fee will be devoted to the objects of the "'Hospital' Convalescent Fund." Any inquiries reaching the office after Monday cannot be answered in "The Mirror" of the current week.

Lower Brittany (Explorer).—It would be delightful for your holiday, only that you must reckon four days lost by the journeys, two for the sea trips and an entire day each way to and from the south. Travelling is very slow. I should go straight to Vannes or Auray, from there visit the Morbihan district, with Quiberon and Lockmariaquer, then on to Quimperlé and Quimper and Douarnenez; from this last you will visit the Point du Raz and the Baie des Trépassés, a most remarkable piece of sea coast. To return you must either go back to Auray or, if time permits, go up to Morlaix and across to St. Malo via Guingamp and Dinan; this route would give you a most excellent idea of general Brittany if you can spare the time, but trains are very slow and connections sometimes hardly deserve the name.

Algiers (Sunny South).—First-class single, £9 12s. 4d.; second-class, £6 14s. 3d. The route is by Dieppe, Paris, and Marseilles. The crossing from Marseilles is often very rough. I should not advise second-class unless it is an imperative necessity. Could you not go second-class on the rail and first on the boat? But so much depends on whether you are a good sailor.

## Figures and Tables

**Fig. 24. f1:**